# GABAergic dysfunction in pediatric neuro-developmental disorders

**DOI:** 10.3389/fncel.2013.00269

**Published:** 2013-12-19

**Authors:** Constance L. Smith-Hicks

**Affiliations:** ^1^Neurology, Kennedy Krieger InstituteBaltimore, MD, USA; ^2^Neurology, Johns Hopkins School of MedicineBaltimore, MD, USA

**Keywords:** PV interneurons, synaptic plasticity, critical period, disease, circuits

## Abstract

The GABAergic system is central to the development and functional maturation of the nervous system. Emerging evidence support the role of GABAergic dysfunction in neuro-developmental disorders. This review presents the molecules and mechanisms that underlie GABA system dysfunction in several neuro-developmental disorders presenting in childhood. The impact on synaptic plasticity, neuronal circuit function and behavior, followed by targeted treatment strategies are discussed.

## Introduction

Neuronal circuits undergo structural refinement in response to changes in experience. Although this ability is present throughout life, there are sensitive periods of elevated plasticity when the brain is capable of intense restructuring of synaptic connections (Wiesel and Hubel, 1963; Knudsen, 2004; Hensch, 2005). These periods of elevated plasticity occur early in postnatal life and are triggered by a unique excitatory—inhibitory (E/I) balance that sets in motion a cascade of molecular events. These molecular events lead to structural changes and closure of the critical period. Critical periods sub-serving the acquisition of different skills occur on different time scales and in multiple brain regions. While they offer tremendous opportunities, they are periods of great vulnerability for the developing brain and critical period dysfunction is linked to several neuro-developmental disorders.

The complexity of neural circuits results in part from the rhythmic inhibitory activity of GABAergic interneurons that synchronize the spiking of local pyramidal cell ensembles, thus maintaining homeostatic levels of activity in the brain (Somogyi et al., 1998; Markram et al., 2004; Somogyi and Klausberger, 2005; Kullmann, 2011). These processes act to enhance the efficiency of information processing by increasing the likelihood of coincident spiking and action potential firing in downstream neurons.

There are several different classes of GABAergic interneurons. Among them are fast-spiking, parvalbumin (PV) positive basket cells which account for 50% of GABAergic interneurons in rodent cortex and are required for the generation of fast rhythmic, synchronous activity (Markram et al., 2004). A single PV+ interneuron innervates hundreds of pyramidal neurons in the mature cortex (Somogyi et al., 1998). Studies have shown that PV interneurons regulate the developmental increase in inhibition that eventually leads to decrease plasticity, and triggers the closure of the critical period in the visual cortex (Heimel et al., 2011). Perturbations in the GABAergic system is linked to alterations in E/I balance, disruption in the critical period, improper development, and function of neural circuits. This leads to aberrant information processing, and cognitive deficits in a variety of neuro-developmental disorders. This review focuses on the GABAergic system dysfunction in Down syndrome, Rett syndrome, Fragile X syndrome, and Autism and therapeutic strategies.

## Down syndrome

Down syndrome (DS) is the most common non-inherited cause of intellectual disability and results from triplication of chromosome 21. The cognitive phenotypes of DS are reproduced in Ts65Dn mice, a model of Down syndrome and are posited to result from imbalance in excitation and inhibition. Several genes involved in the GABAergic systems are present on chromosome 21 and are triplicated in DS and the Ts65Dn mice. These include Olig1/2 and Grik2. *Olig1* and *Olig2 are over*-expressed in the medial ganglionic eminence (MGE) of Ts65Dn brain at E14.5 (Chakrabarti et al., 2010). There are more *Olig2*^+^ cells in the Ts65Dn MGE compared to euploid littermates at E13.5 and E14.5 when cohorts of cortical interneurons arise. *Olig1/Olig2* over-expression is thought to be the genetic basis of the interneuron over-production in the Ts65Dn brain. Reducing the gene dosage of *Olig2* in Ts65Dn mice rescued the interneuron over-production phenotype, and normalized the frequency of spontaneous inhibitory postsynaptic currents (sIPSC). G-protein coupled inward-rectifying potassium (GIRK2) containing channels are over-expressed in Ts65Dn mice (Harashima et al., 2006). These channels are activated by agonists at Gi/o coupled receptors such as the GABAB receptor (Sodickson and Bean, 1996). Their pattern of expression mirrors the expression of GABAB receptors and Grik2, and GABAB receptors are more intensely expressed in the stratum lacunosum moleculare (SLM) when compared to the other layers of the CA1 (Sloviter et al., 1999; Lopez-Bendito et al., 2004; Harashima et al., 2006). The SLM is expected to have larger synaptically evoked inhibitory currents (Pham et al., 1998) and likely serve as primary modulators of the direct perforant pathway inputs into CA1, thus playing important roles in learning and memory (Sybirska et al., 2000; Brun et al., 2002). Elevated GABAB receptor dependent GIRK channel current density was observed in CA1 of Ts65Dn mice and occurred without differences in GABA release probability was (Best et al., 2007, 2012). In addition, the ratio of monosynaptic GABAB/GIRK to GABAA currents was significantly elevated in Ts65Dn mice upon SLM stimulation in the absence of changes in GABAA subunit expression or function. The experiments described by Best et al. (2012) attribute the altered GABAergic transmission in area CA1 of Ts65Dn mice to increase function of the GABAB/GRIK complexes. In contrast, data from Kleschevnikov et al., demonstrates enhanced GABAA and GABAB receptor-mediated evoked IPSCs in the DG granule cells with decrease in paired-pulse ratio suggesting an increase in presynaptic release (Kleschevnikov et al., 2004). Together these data suggest that there are region specific mechanisms underlying GABAergic signaling deficits within theTs65Dn hippocampus.

Several groups show a decrease in the number of excitatory synapses and a relative increase in the inhibitory synaptic markers in the cortex and hippocampus of adult Ts65Dn mice (Belichenko et al., 2004, 2009; Perez-Cremades et al., 2010). Consistent with these findings the number of inhibitory interneurons is increased in Ts65Dn mice (Chakrabarti et al., 2010). Experiments designed to examine the functional relevance of the increase in synaptic markers show that the rate of sIPSC is higher in the pyramidal cell layer of CA1 in Ts65Dn mice. Moreover, there were no differences in the frequency of mIPSC or in the paired pulse ratio. This finding is consistent with an increase in the number of functional interneurons and is inconsistent with an increase in number of inhibitory synapses or enhanced probability of GABA release in CA1 (Chakrabarti et al., 2010; Best et al., 2012; Mitra et al., 2012). Developmental assessment of the IPSC shows a transient increase in evoked inhibition and mIPSC in Ts65Dn mice during the second postnatal week but not in the first or third postnatal week, when stimulation is performed in the stratum radiatum or pyramidale (Mitra et al., 2012). This transient disruption in inhibitory synaptic transmission occurs during the critical period of neuronal network development and could contribute to later network dysfunction seen in adult mice.

Enhanced GABA-mediated inhibition in several hippocampal sub-regions is associated with a marked reduction in long-term potentiation (LTP) in the CA1 and DG. Several groups have rescued LTP upon acute or chronic treatment of Ts65Dn hippocampus with GABA antagonist (Siarey et al., 1997; Kleschevnikov et al., 2004; Costa and Grybko, 2005; Fernandez and Garner, 2008). Consistent with these findings and a role for GABA in learning and memory, modulators of GABA receptor function have been used to rescue cognitive phenotypes in the Ts65Dn mice. The non-competitive GABAA antagonist pentylenetetrazole (PTZ), and the GABA antagonist picrotoxin and bilobalide normalized cognitive function measured by object recognition (Fernandez et al., 2007), while chronic administration of PTZ rescued the performance of Ts65Dn mice in Morris water maze (Rueda et al., 2008). Moreover, RO4938581 (3-bromo-10-(difluoromethyl)-9H-benzo_f_imidazo[1,5-a][1,2,4] triazolo[1,5-d][1,4]diazepine), a selective GABA A _5 NAM, rescued hippocampal LTP and hippocampal dependent cognitive deficits in Ts65Dn mice (Martinez-Cue et al., 2013), and the GABA-A-benzodiazepine receptor inverse agonist selective for the a5-subtype (a5IA) restored learning and memory in novel object and Morris water maze in Ts mice (Braudeau et al., 2011). GABAB receptor antagonism relevant to DS since the GABAB receptor antagonist CGP55845 improved contextual memory in fear-conditioning task in Ts65Dn mice (Kleschevnikov et al., 2012). Clinical trials are underway to study the effect of GABA antagonism on cognitive phenotypes in DS.

## Rett syndrome

Rett syndrome (RS) is the leading genetic cause of intellectual disability in girls. It is caused by mutations in the transcriptional repressor methyl-CpG-binding protein 2 (MeCP2). The MECP2 protein regulates gene expression by DNA methylation and MeCP2-mutant mice recapitulate many of the phenotypes RS. These mice, like girls with RS appear phenotypically normal early during development and then undergo severe regression with resulting impairment in respiratory function, loss of hand use, impairment in language, and motor function. Several studies suggest that an imbalance in excitatory and inhibitory neurotransmission contributes to the behavioral abnormalities in RS (Dani et al., 2005; Asaka et al., 2006; Moretti et al., 2006).

In early postnatal life before respiratory abnormalities become apparent, a shift in the E/I balance toward excitation is seen in the ventrolateral medulla of MeCP2 null mice. This shift results from a disruption in both presynaptic and post-synaptic mechanisms of the excitatory and inhibitory pathways (Medrihan et al., 2008). This is in contrast to the increase in inhibitory and reduction in excitatory inputs to L5 pyramidal neurons in the somatosensory cortex during the second postnatal week, also before onset of overt symptoms. The direction of the shift is brain region specific and this is readily apparent in thalamic networks. In the absence of MeCP2, a reduction in frequency of mIPSC in the ventrobasal complex (VB) of the thalamus is observed during the first through third postnatal week but an increase in frequency of mIPSC was observed from the second through third postnatal week in the reticular thalamic nucleus (RTN) (Zhang et al., 2010). A further example of brain region specific change is the shift of E/I balance in favor of inhibition reported in the visual cortex of MeCP2 null mice. Parvalbumin (PV) levels and the number of PV positive interneurons were increased, while the mRNA of the calcium-binding proteins, calretinin, and calbindin were reduced. In addition, perisomatic boutons were increased in MeCP2 KO mice and this increase was evident at the time of eye opening. PV+ interneurons synapse onto the soma and proximal dendrites of principal cells to control their excitability and adjust the gain of their integrated synaptic response (Markram et al., 2004; Atallah et al., 2012). Maturation of these PV circuits in visual cortex is modulated by sensory experience. Dark-rearing from birth (DR) specifically reduces perisomatic inhibition (Katagiri et al., 2007; Sugiyama et al., 2008) and delays onset of the heightened period of visual cortical plasticity. Dark rearing at P30 in the absence of MeCP2 was sufficient to rescue PV-cell hyper-connectivity and improve visual acuity to near WT levels in the adult MeCP2 null mice. Restoration of visual acuity was associated with normalization of PV levels, a reduction in the number of perisomatic boutons and reduction in spontaneous firing rates of MeCP2 KO mice to that of control WT cells (Durand et al., 2012).

The experiments described above clearly demonstrate a critical role for MeCP2 in regulation of GABAergic transmission. The cellular and molecular mechanisms underlying MeCP2-mediated regulation of GABAergic transmission are diverse and highly circuit dependent. Several studies show that BDNF regulates the formation and maturation of GABAergic synapses (Huang et al., 1999; Marty et al., 2000; Hong et al., 2008), and the reduction of BDNF in single cortical neurons caused a reduction in the frequency of mIPSC without change in peak amplitude (Kohara et al., 2007). Indeed, BDNF is regulated by MeCP2 in an activity dependent manner (Zhou et al., 2006) and BDNF levels are reduced in MeCP2 null mice (Chang et al., 2006; Wang et al., 2006). Intriguingly, MeCP2 null mice that are raised in an enriched environment (EE) starting at P10 show increase in BDNF levels and normalization of LTP and spatial memory when measured in adult mice (Lonetti et al., 2010). The normalization of synaptic plasticity and behavior occurs in the context of an increase in the density of excitatory synapses in the somatosensory cortex with no changes in the density of inhibitory synapses (Lonetti et al., 2010). EE have previously been shown to cause a functional reduction in cortical inhibition (Sale et al., 2007), whether a similar mechanism contributes to the amelioration of behavioral and plasticity deficits in MeCP2 null mice reared in an EE is not known. These data suggest that the interplay between MeCP2 and BDNF on the balance between E/I is complex and can be further modulated by environmental context.

## Fragile X syndrome

Fragile X syndrome (FXS) is the most common single gene cause of intellectual disability and autism (Kaufmann et al., 2004; Hagerman et al., 2005). It results from loss of function mutations in the *fmr1* gene that encodes FMRP (Verkerk et al., 1991; O'Donnell and Warren, 2002). The FX phenotypes of cognitive impairment, hypersensitivity to sensory stimuli, epilepsy, and characteristics of autism are recapitulated in the *Fmr1* knockout (KO) mouse a model of FXS (Musumeci et al., 2000; Nielsen et al., 2002; Spencer et al., 2005; Brennan et al., 2006). An imbalance in excitation and inhibition is posited to be central to the etiologic mechanisms of Fragile X.

Several studies have suggested a role for disruption of the GABAergic system in FXS and report abnormal GABA-mediated transmission in FX KO mice. Impairment in the excitatory inputs onto fast spiking (FS) interneurons and impairment in group 1 mGluR activation of low-threshold-spiking (LTS) interneurons have been reported in FX mice (Gibson et al., 2008; Paluszkiewicz et al., 2011). The inhibitory output of the FS and LTS interneurons typically increase in response to network activation (Kapfer et al., 2007; Silberberg and Markram, 2007), thus making them well positioned to dynamically control cortical network function. In FX mice this disruption in activation of FS and LTS is implicated in cortical hyperexcitability manifesting as altered UP States and inhibitory network dysfunction in layerII/III of the somatosensory cortex.

GABAA receptors are heteropentamers, and their subunit composition is an important determinant of inhibitory transmission, subcellular localization, and sensitivity to clinically relevant compounds (Hevers and Luddens, 1998; Sieghart and Sperk, 2002; Rudolph and Mohler, 2006). Expression studies reveal that mRNAs that encode the GABAA α1, α3, α4 receptor subunits are decreased in adult Fmr1 KO mice (El Idrissi et al., 2005; Gantois et al., 2006; D'Hulst et al., 2009). Protein levels of GABAA receptor α1, β2, and δ subunits are transiently down regulated at postnatal day 12 and all but GABAA β2 subunits were restored to normal levels in adult mice. GABAA receptor α1 subunit is the most abundant of all GABAA receptor subunits (Mohler, 2007; Olsen and Sieghart, 2009), they account for about half of all GABAA subunits and are widely expressed in the CNS. The reduction of GABAA receptors at P12 is posited to cause a reduction in excitation in the second week of postnatal development at a time when GABAergic synaptic activity is thought to play a critical role in the maturation of functional inhibitory synapses (Ben-Ari, 2002; Cancedda et al., 2007). Thus, the down regulation of the GABAA receptor subunits at P12 may lead to structural alterations of synapses that result in the characteristic presence of immature dendritic spines in FXS.

Presynaptic increases and decreases in the expression of the GABA-synthesizing enzyme GAD have been reported in *Fmr1* KOs (El Idrissi et al., 2005; D'Hulst and Kooy, 2009; Adusei et al., 2010; Olmos-Serrano et al., 2010). The direction of the change is due in part to the brain region examined. Olmos-Serrano and colleagues report that GAD65/67 protein expression is reduced in the basolateral amygdala, and this finding is complemented by decreased vesicular GABA levels. In contrast GAD65/67 levels were increased in the cortex, hippocampus, brainstem, and diencephalon (El Idrissi et al., 2005; Adusei et al., 2010). Transient down regulation of the catabolic enzymes GABA-T and succinic semialdehyde dehydrogenase are reported in the forebrain of FMRP null mice at P12 (Adusei et al., 2010) and may compensate for the decrease levels of GABAA receptor subunits to effectively increase GABAergic transmission by increasing GABA levels.

FMRP KO mice exhibit variable and region specific disruptions in functional inhibitory transmission in behaviorally relevant brain regions. Inhibitory dysfunction in the basolateral amygdala is characterized by reductions in the frequency and amplitude of spontaneous IPSCs and changes in IPSC kinetics. These findings are consistent with report of decreased GABAA receptor subunit, GAD expression, vesicular GABA, and impaired GABA release (Olmos-Serrano et al., 2010). In contrast, striatal neurons demonstrate an increase in basal inhibitory transmission driven by increased GABA release as show by the increase in the frequency of sIPSCs, mIPSCs, and reduction in PPR of evoked IPSCs (Centonze et al., 2008).

GABAergic dysfunction in FMRP KO mice is mediated by presynaptic and post synaptic mechanisms that are brain region and receptor subtype dependent. With this in mind preclinical and clinical trials have examined the therapeutic relevance of GABAA and GABAB receptors in FXS. THIP (gaboxadol), a GABAA receptor agonist, that acts upon tonic GABA receptors significantly attenuated hyperactivity and prepulse inhibition in *Fmr1* KO mice, but did not reverse the deficits in cued fear or startle response (Olmos-Serrano et al., 2011). Activation of the GABAB receptor with STX209 (arbaclofen, R-baclofen) corrected synaptic abnormalities, reduces protein synthesis, correct enhanced AMPAR trafficking, and suppressed audiogenic seizures in FMRP KO mice (Pacey et al., 2009; Henderson et al., 2012). Moreover, STX209 when used in a clinical trial showed improved social function in patients with FXS (Berry-Kravis et al., 2012). Additional clinical trials are underway to test the ability of STX209 to reduce aggression and irritability in FXS. The variable mechanism by which GABA dysfunction contributes to the FX phenotype poses interesting challenges to treatment that may require the use of combination therapies.

## Autism

Autism Spectrum Disorder is postulated to result in part from deficient GABA neurotransmission. Reduced GABAA receptors and benzodiazapine binding sites are reported in the hippocampus and cingulate gyrus from neuropathological samples from individuals with autism (Blatt et al., 2001; Oblak et al., 2009, 2010). Consistent with the findings in autopsy samples, direct measurement of GABAA receptors and GABA concentration in the living brain of children with ASD using SPECT (Single Photon Emission Computed Tomography) and [1H]MRS showed a reduction in GABAA receptor levels and GABA concentration in the frontal cortex (Harada et al., 2011; Mori et al., 2012). Examination of GABAA receptor levels in the brains of adults with ASD, using Positron Emission Tomography (PET) and the benzodiazepine receptor PET ligand [11C]Ro15-4513, showed a reduction in 11C] Ro15-4513 binding in the amygdala and nucleus accumbens (Mendez et al., 2013).

Converging on neuropathological and imaging data, genetic studies reveal a role for GABAergic dysfunction in ASD. Microduplications/deletions of the 15q11- q13 chromosomal locus, known to contain GABAA a5, b3, and g3 subunits, have been observed in some individuals with ASD (Cook et al., 1998; Menold et al., 2001; Buxbaum et al., 2002; Shao et al., 2003; McCauley et al., 2004; Sebat et al., 2007). Duplication of this locus is expected to result in over expression of the GABAA receptor subunits, however, *in vitro* studies using human neuronal cell line carrying the 15q duplication showed a reduction in GABRB3 expression (Meguro-Horike et al., 2011). The association of genetic polymorphisms in the *DLX1/2* genes provides additional support of the GABAergic model for autism (Liu et al., 2009). In addition, transciptomic analysis of autopsy samples from autistic brains found reduced cortical mRNA expression of genes that are highly expressed in PV+ interneurons (Voineagu et al., 2011). Together these studies provide strong convergent evidence for an inhibitory deficit in autism.

## Conclusion

The volume of evidence in support of GABAergic dysfunction in neuro-developmental disorders is overwhelming. Although the mechanism and manifestation of GABA dysfunction is disease and brain region/circuit specific, current evidence supports impaired GABA transmission during the critical period followed by GABAergic dysfunction in the adult animal. Current efforts to treat the disorders discussed have had variable results. One approach worthy of consideration involves reintroducing a period of elevated plasticity in the adult animal. Such approach has major hurdles. It requires a detailed understanding of GABAergic dysfunction, methods for reopening the critical period sub-serving the acquisition of different skills and the impact of such manipulations on other brain regions. Much can be learned from efforts to treat amblyopia by re-opening the critical period. Several groups have reintroduced juvenile plasticity in the adult brain of rodents by directly removing the perineuronal nets (PNN), a structural barrier to plasticity thus enabling recovery from amblyopia (Pizzorusso et al., 2002; Sale et al., 2007; Soleman et al., 2013). Manipulations that adjust the E/I balance either by direct transplantation of embryonic GABA precursor cells into the postnatal brain (Southwell et al., 2010) or the endogenous release of serotonin through chronic treatment with fluoxetine have opened a second critical period that restores visual function in amblyopic adult rats (Maya Vetencourt et al., 2008). Chronic administration of fluoxetine mediates its effect through the reduction of intracortical inhibition and increase in BDNF levels (Maya Vetencourt et al., 2008) possibly allowing activation of plasticity genes and modulation of neuronal circuits involved in ocular dominance. Environmental enrichment through enhanced sensory-motor stimulation rescued spatial learning and visual acuity in adult Ts65Dn mice (Begenisic et al., 2011). These changes occur in association with a reduction in GABA release and normalization of hippocampal LTP. These findings are consistent with previous reports that show reduced cortical inhibition and increased neuronal plasticity in the brains of adult rats exposed to an EE (Sale et al., 2007). Moreover, the restoration of near normal visual acuity in MeCP2 null mice upon dark rearing at P30 is encouraging, and suggests that reopening the critical period maybe a therapeutic option for neuro-developmental disorders. There has been some success at restoring vision in human adults with amblyopia (Bavelier et al., 2010) but the need to better understand the extent to which sensitive periods can be safely and noninvasively recapitulated remains an exciting challenge.

### Conflict of interest statement

The author declares that the research was conducted in the absence of any commercial or financial relationships that could be construed as a potential conflict of interest.
